# Phineas Gage's great legacy

**DOI:** 10.1590/1980-57642020dn14-040013

**Published:** 2020-12

**Authors:** Ricardo Vieira Teles

**Affiliations:** 1 Faculty of Medicine, Universidade Federal de Goiás – Goiânia, GO, Brazil.

**Keywords:** Phineas Gage, behavioral symptoms, history, Phineas Gage, sintomas comportamentais, história

## Abstract

The case of Phineas Gage is an integral part of medical folklore. His accident still causes astonishment and curiosity and can be considered as the case that most influenced and contributed to the nineteenth century's neuropsychiatric discussion on the mind-brain relationship and brain topography. It was perhaps the first case to suggest the role of brain areas in determining personality and which specific parts of the brain, when affected, can induce specific mental changes. In addition, his case contributed to the emergence of the scientific approaches that would later culminate in psychosurgery. Gage is a fixed element in the studies of neurology, psychology, and neuroscience, having been solidified as one of the greatest medical curiosities of all time, deserving its prominence.

## RESUMO.

O caso de Phineas Gage é parte integrante do folclore médico. Seu acidente ainda causa espanto e curiosidade, e pode ser considerado como o caso que mais influenciou e contribuiu para a discussão neuropsiquiátrica do século XIX sobre a relação mente-cérebro e topografia cerebral. Foi talvez o primeiro caso a sugerir o papel de áreas cerebrais na determinação da personalidade e que partes específicas do cérebro, quando afetadas, podem induzir mudanças mentais específicas. Além disso, seu caso contribuiu para o surgimento de abordagens cientificas que culminariam posteriormente na psicocirurgia. Gage é um elemento fixo nos estudos de neurologia, psicologia e neurociências, tendo sido solidificado como uma das grandes curiosidades médicas de todos os tempos que merece seu destaque.

## THE ACCIDENT

Gage, a 25-year-old male, 1.70 m in height and weighing approximately 70 kg, was employed in railroad construction at the time of the accident. As the company's most capable employee, with a well-balanced mind and a sense of leadership, he was directing a rock-splitting workgroup while preparing the bed of the Rutland & Burlington Railroad south of Cavendish, Vermont, USA. At 4:30 PM on September 13, 1848, he and his group were blasting a rock, and Gage was assigned to put gunpowder in a deep hole inside it.^1^

The moment he pressed the gunpowder into the hole with a bar, the friction caused sparks, and the powder exploded. The resulting blast projected the meter-long bar, which was 3.2 cm in diameter and weighed about 6 kg, through his skull at high speed. The bar entered his left cheek, destroyed his eye, passed through the left front of the brain, and finally completely left his head at the top of the skull on the right side. Gage was thrown on his back and had some brief convulsions, but he woke up and spoke in a few minutes, walked with a little help, and sat in an ox cart for the 1.2-km trip to his quarters.^1^

In the city about 30 minutes after the accident, Doctor Edward H. Williams arrived to provide medical care. Gage had lost a lot of blood, and his following days were quite difficult.^1^ The wound became infected, and Phineas was anemic and remained semicomatose for more than two weeks. He also developed a fungal infection in the exposed brain that needed to be surgically removed. His condition slowly improved after doses of calomel and beaver oil. By mid-November he was already walking around the city.^2^

## THE CONSEQUENCES

**Figure 1:**
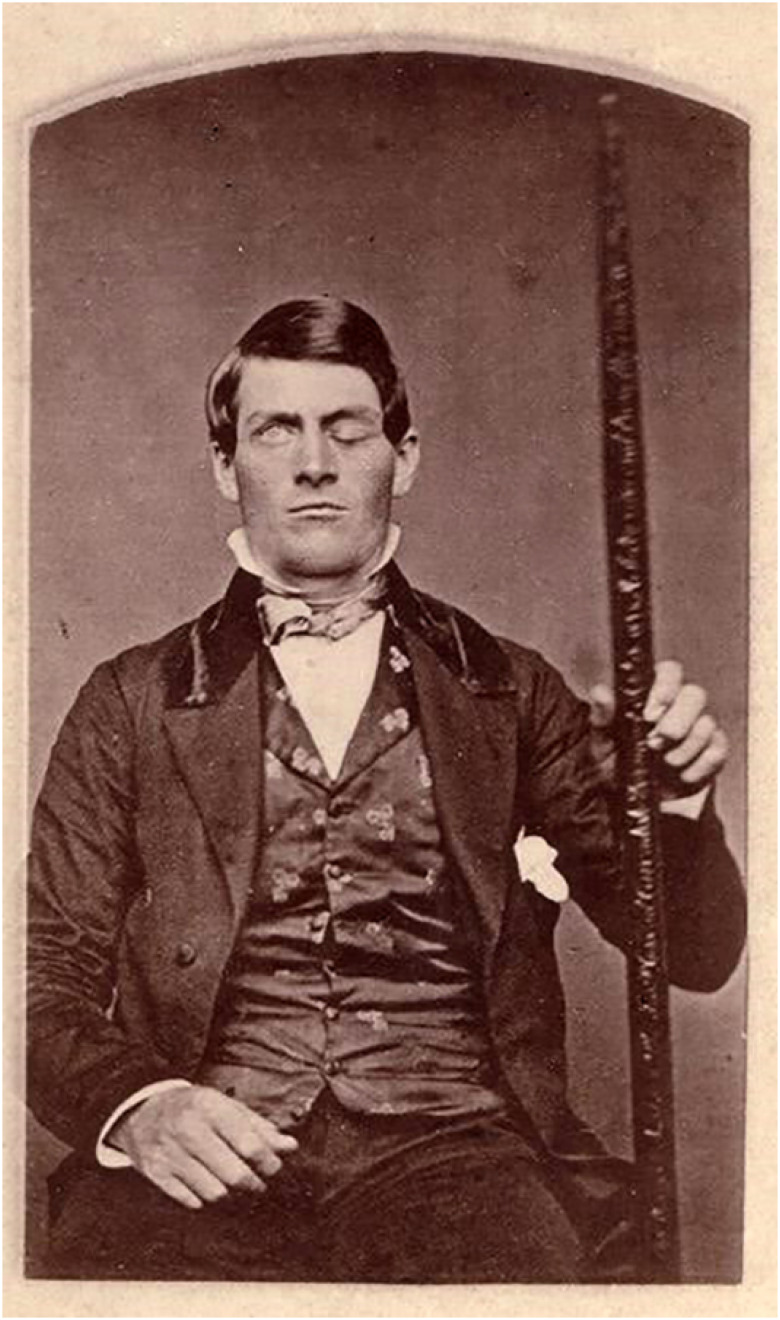
Gage holding the iron bar that injured him.

Source: Phyllis Gage Hartley/Creative Commons.

For three weeks after the accident, the wound was treated by doctors. During this time, he was assisted by Dr. John Harlow, who covered the head wound and then reported the case in the Boston Medical Surgery Journal. In November 1849, invited by the professor of surgery at Harvard Medical School, Henry Jacob Bigelow, Harlow took Gage to Boston and introduced him to a meeting of the Boston Society for Medical Improvement (Figure 1).^3^

In his reports, Harlow described that the physical injury profoundly altered Gage's personality. Although his memory, cognition and strength had not been altered, his once gentle personality slowly degraded. He became a man of bad and rude ways, disrespectful to colleagues, and unable to accept advice. His plans for the future were abandoned, and he proceeded without thinking about the consequences.^4^ And here was the main point of this curious story: Gage became irritable, irreverent, rude and profane, aspects that were not part of his way of being. His mind had changed radically. His transformation was so great that everyone said that “Gage is no longer himself.”^5^

**Figure 2:**
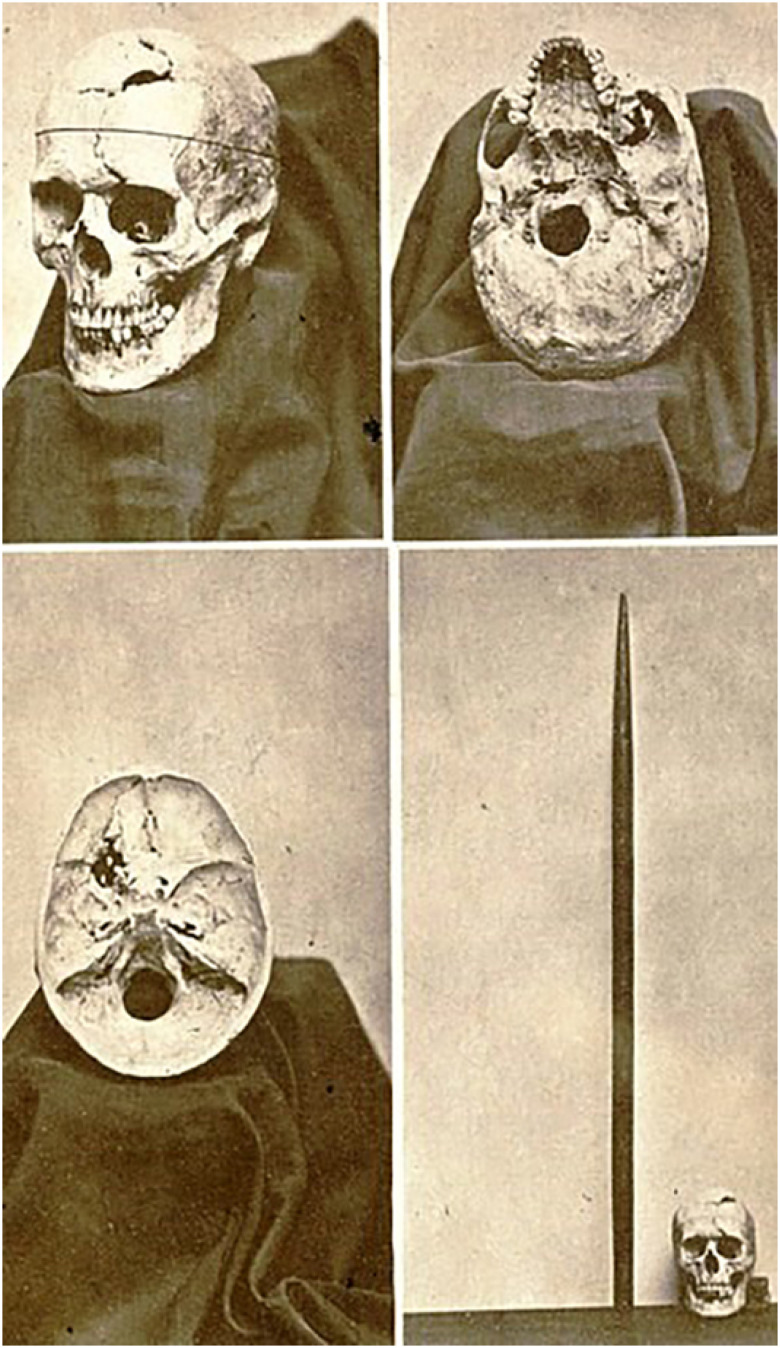
Gage's exhumed skull and the iron bar, 1870.

Source: Image via J.B.S. Jackson/A Descriptive Catalog of the Warren Anatomical Museum.

As a result of this personality change, he was fired for indiscipline and could no longer hold a steady job. He became a circus attraction and even tried life in Chile, later returning to the United States. However, there is something still little known about Gage: his personality changes lasted for about four years, slowly reverting later. As a proof of this, he worked as a long-haul driver in Chile, a job that required considerable planning and focus skills. He died on May 21, 1861, 12 years after the accident, from an epileptic seizure that was almost certainly related to his brain injury. He was not submitted to an autopsy, but his mother, after exhumation of the body, donated his skull and iron rod at the request of Dr. Harlow, which, in turn, sometime later donated them to Harvard University (Figure 2).^1^

## THE LEGACY

Gage's case is considered to be one of the first examples of scientific evidence indicating that damage to the frontal lobes may alter personality, emotions and social interaction.^6^ Prior to this case, the frontal lobes were considered silent structures, without function and unrelated to human behavior. Scottish neurologist, David Ferrier, was motivated by this fact to investigate the role of frontal lobes in brain function. Ferrier removed the frontal lobes in monkeys and noted that there were no major physiological changes, but the character and behavior of the animals were altered.^7^

Knowledge that the frontal lobe was involved with emotions continued to be studied. The surgeon Burkhardt in 1894 performed a series of surgeries in which he selectively destroyed the frontal lobes of several patients in whom he thought might control psychotic symptoms, being the modern prototype of what was later known through Egas Moniz as psychosurgery.^7^ Today, it is well understood that the prefrontal cortex of the brain controls the organization of behavior, including emotions and inhibitions.

Folkloric as it may be, but nonetheless remarkable, the contribution of Phineas Gage's case should not be overlooked, as it provided scientists the baseline for the promotion of studies in neuropsychiatry, and a source of inspiration for world medicine.^8^ In 2012, a team of neuroscientists used computed tomography of Gage's skull with typical brain MRI scans to show how the Gage brain connection could have been affected.^9^ And it is not just the researchers who keep coming back to Gage. Medical and psychology students still learn about Gage from their history lessons. Neurosurgeons and neurologists still sometimes use Gage as a reference when evaluating certain cases.^10^ The final chapter of his life also offers us a thought-provoking learning about cases of massive brain damage, showing us that rehabilitation may be possible.^11^

Therefore, Gage — inadvertently — made a huge contribution to neurology in several areas, including the study of brain topography in behavioral disorders, the development of psychosurgery, and finally the study of brain rehabilitation. Also, Gage's case had a tremendous influence on early neuropsychiatry. The specific changes observed in his behavior pointed to theories about the localization of brain function and correlated with cognitive and behavioral sequelae, thereby acquainting us with the role of the frontal cortex in higher-order actions such as reasoning, behavior and social cognition. In those years, while neuropsychiatry was in its infancy, Gage's extraordinary story served as one of the first pillars of evidence that the frontal lobe is involved in personality, which helped solidify his remarkable legacy in world medical history.
